# The psychopathological and psychosocial outcome of early-onset schizophrenia: Preliminary data of a 13-year follow-up

**DOI:** 10.1186/1753-2000-2-6

**Published:** 2008-02-27

**Authors:** Andreas Reichert, Susanne Kreiker, Claudia Mehler-Wex, Andreas Warnke

**Affiliations:** 1 Department of Child & Adolescent Psychiatry, Psychosomatic Medicine, and Psychotherapy, University of Wuerzburg, Germany; 2 Department of Child & Adolescent Psychiatry and Psychotherapy, University of Ulm, Germany

## Abstract

**Background:**

Relatively little is known about the long-term psychopathological and psychosocial outcome of early-onset schizophrenia. The existing literature describes more severe courses of illness in these patients compared with adult-onset schizophrenia. This article reports preliminary data of a study exploring the outcome of early-onset schizophrenia 13.4 years (mean) after first admission. Predictors for interindividual outcomes were investigated.

**Methods:**

We retrospectively assessed 27 former patients (mean age at first admission 15.5 years, SD = 2.0) that were consecutively admitted to the Department of Child and Adolescent Psychiatry at the University of Wuerzburg between 1990 and 2000. A multidimensional approach was chosen to assess the outcome consisting of a mail survey including different questions about psychopathological symptoms, psychosocial parameters, and standardized self-reports (ESI and ADS).

**Results:**

Concerning the psychopathological outcome, 22.2% reported having acute schizophrenic symptoms. Almost one third (30.8%) described symptoms of depression and 37.0% reported having tried to commit suicide or seriously thought about it. 77.8% of the former patients were still in outpatient treatment. Compared to the general population, the number of patients without a school graduation was relatively high (18.5%). Almost half of participants still live with their parents (48.1%) or in assisted or semi-assisted living conditions (33.3%). Only 18.5% were working in the open market.

**Conclusion:**

Schizophrenia with an early onset has an unfavourable prognosis. Our retrospective study of the psychopathological and psychosocial outcome concludes with a generally poor rating.

## Background

Schizophrenia is one of the most deteriorating psychiatric disorders. In the words of Carpenter [1] "this illness strikes at the very heart of what we consider the essence of the person. Yet, because its manifestations are so personal and social, it elicits fear, misunderstanding, and condemnations in society instead of sympathy and concern". In the investigation of the course and outcome of this disorder, existing studies examined large populations of adult schizophrenic patients. These follow-up studies often found one third with a good outcome, one third with a moderate outcome and one third with a poor outcome [2,3]. Häfner and colleagues [4] showed that two thirds of a sizeable sample of inpatients had repeated inpatient treatment because of schizophrenia ten years after first admission (mean number of inpatient treatments: women: 3.4, men: 3.7). Furthermore, a meta-analysis of 320 follow-up studies on schizophrenia beginning in adulthood revealed a 'good' outcome in only 40% of the cases [5]. In their ABC-study, Häfner et al. [6] reported similar results. They reassessed 107 former patients after 12 years and found that 44% had no symptoms of schizophrenia during the nine months before reassessment.

Compared with the literature on adult schizophrenia, the number of follow-up studies of schizophrenia with a beginning in childhood or adolescence is relatively little. Authors often distinguish between early onset schizophrenia (EOS) under the age of 18 years and a very early onset under the age of 14 years (VEOS). Besides the small number of studies, follow-up investigations of VEOS and EOS often have a minor sample size, as the incidence and prevalence of VEOS and EOS is very little. Analyzing studies about the outcome of schizophrenia beginning in childhood or adolescence [7-10], one infers that the course and outcome is less favourable than in adult schizophrenic psychoses. Alaghband-Rad [11], for example, states that childhood-onset schizophrenia (in his study before age 12 years) represents a more malignant form of the disorder. Another line of research by Hollis [12,13] shows that premorbid social, motor and language impairments are especially marked in VEOS and EOS compared to other forms of psychiatric disorders with early onset. Overall, it appears that schizophrenic adults were more likely to achieve periods of improvement, a higher level of psychosocial functioning and a better overall outcome.

Werry et al. [7], for example, investigated 30 former schizophrenic patients 7 to 17 years after their first inpatient treatment. Complete recovery was noted for only 23% of their patients at follow-up. Therefore, the authors described EOS as a chronic or relapsing disorder. As a possible predictive factor, they mentioned the premorbid adjustment level. In comparison, Gillberg and his colleagues [8] found even worse results in their follow-up study. Only 13% of the 23 former patients showed a good outcome 11 to 17 years after follow-up.

Asarnow et al. [14] assessed a sample of 18 patients diagnosed as adolescent schizophrenia. They reported a good level of psychosocial functioning in only 28% of the cases. 17% showed a deteriorating course and 28% only minimal improvement. Another follow-up study by Maziade and his colleagues [15] also points towards a negative course of EOS. In their sample of 40 patients, only 5% of patients achieved a total recovery after a mean follow-up interval of 14.8 years. In 34% of the cases, the outcome was described as poor, in 40% as very poor.

In contrast to the results of the already mentioned studies, relatively high rates of complete and partial remission in the long-term course of schizophrenia were reported by Eggers and Bunk [9]. They reassessed 44 inpatients after a long follow-up interval of 42 years. 25% were completely, 25% partially, and 50 percent were poorly remitted. As possible risk factors, they discussed the age at onset of the disorder and the type of onset (acute versus chronic).

In a follow-up study 10 years after the first psychotic episode, Lay et al. [16] re-examined 65 children (VEOS) and adolescents (EOS) with schizophrenia. Serious social disability was found in 66% of patients; whereas 14% had an obvious and 20% had no or minimal social disability. In this study, a longer duration of inpatient stay was associated with a lower level of functioning at follow-up.

In a recent study, Fleischhaker et al. [10] reassessed 81 children (VEOS) and adolescents (EOS) with schizophrenia 9.5 years after the onset of the disorder. Very good or good outcome was found in 20% of the patients. 38% showed a moderate outcome whereas 42% had a very poor outcome or gross impairment. Premorbid adjustment was the best predictor of outcome in their sample. They also investigated psychosocial factors like education, living conditions and occupational situation at follow-up, showing that only 29% were employed on a non-sheltered basis and half of the patients lived in assisted-living establishments.

Another current study by Röpcke and Eggers [17] investigated the psychopathological and psychosocial outcome after a mean followup of 15 years. They found a higher percentage of moderate outcome (56%) and a comparable number of patients with a poor outcome (36%). The best predictor of global psychopathological and psychosocial outcome was type of onset.

Remschmidt and colleagues [18] recently examined the outcome of VEOS after a mean time span of 42 years. The diagnosis of schizophrenia was confirmed by consensus analysis in only 50% of the original sample of 76 patients. The majority showed a poor (60%) or moderate (24%) global outcome. A high percentage of the former patients failed to graduate from any school (74%) and were unemployed (71%). Again, this study emphasized the poor psychopathological and psychosocial outcome of childhood-onset schizophrenia.

A short overview about some results of the mentioned follow-up studies is given in Table 1.

**Table 1:** Overview about follow-up studies on VEOS and EOS

**Study**	**Number of reassessed patients**	**Age at onset (range in years)**	**Follow-up interval (years)**	**Outcome (rounded values)**
Werry et al. [7]	30	7–17	1–16	*Status of disorder at follow-up:* 23% quiescent 13% subchronic 64% chronic
				*Living situation:* 7% independent 13% semidependent 67% dependent (13% dead)
				*Episodes since index:* 3% none 7% one/two 90% two+
Gillberg et al. [8]	23	13–19	11–17	*Overall outcome:* 13% good 9% intermediate 78% extremely poor
Asarnow et al. [14]	18	6–13	2–7	*Psychosocial functioning:* 28% good 28% moderate improvement 28% minimal improvement 17% deteriorating course
Eggers & Bunk [9]	44	6–14	42	*Overall outcome:* 25% complete remission 25% partial remission 50% no remission
Lay et al. [16]	65	11–17	12	*Social disability:* 20% none or minimum 14% obvious 30% serious 31% very serious 5% maximum dysfunction
				*Sources of income:* 25% self 39% parents/spouse 37% public assistance
				*Marital status:* 91% single 5% married 5% divorced
Fleischhaker et al. [10]	81	16 (mean)	10	*Outcome (Global Assessment Scale, GAS):* 20% good 38% moderate 42% poor
				*Employment:* 38% clinical setting 14% semisheltered basis 29% nonsheltered basis 18% did not work
				*Depressive symptoms:* 21% severe symptoms 19% moderate symptoms
Röpcke & Eggers [17]	39	16 (mean)	15	*Severity of symptoms:* 8% full remission 56% moderate outcome 36% poor outcome
				*Employment:* 5% clinical setting 20% regular occupation 36% sheltered basis 31% did not work
				*Marital status* 69% single 31% partner/family
Remschmidt et al. [18]	38	5–14	42	*Outcome (GAS):* 16% good 24% moderate 60% poor
				*Employment:* 71% unemployed 5% employed 24% receiving a pension
				*Marital status:* 74% single or death 18% married 8% divorced or widow

Because of the small number of studies on EOS and the small sample sizes in the majority of the studies, important questions remain concerning the long-term course, outcome and predictive factors of this disorder. The present study will report on the preliminary data of a follow-up examination with a mean time span of 13 years. The aim of our study is to investigate course, psychopathological and psychosocial outcome of EOS. In addition, we will try to identify risk factors or predictors for the long-term outcome. Therefore, former schizophrenic patients were identified and were asked to fill out a questionnaire (mail survey). According to Häfner and an der Heiden [19], we followed a multidimensional approach of assessing psychopathological and psychosocial outcome. To our knowledge, this is the first study on the outcome of EOS that also used specific, high standardized instruments in a mail survey to examine positive and negative symptoms. In the following sections, we will present preliminary data of the mail survey.

## Methods

### Original Sample

The sample consisted of patients with a clinical diagnosis of a schizophrenic or schizoaffective disorder (n = 86) consecutively admitted to the Department of Child and Adolescent Psychiatry at the University of Wuerzburg between 1990 and 2000. We also included patients with the diagnosis of a schizoaffective disorder, since previous studies showed no substantial differences in the outcome of this disorder compared to schizophrenic patients [20].

Specifically, we included former patients in our schizophrenia sample that met the following criteria:

• They were consecutively admitted to our clinic and were treated as schizophrenia or schizoaffective patients according to the ICD-9- (295) or ICD-10-criteria (F20, F25) for more than one day.

• They were younger than 18 years old and received their first inpatient treatment due to schizophrenia.

A retrospective diagnostic evaluation was carried out by two experienced clinicians checking the patients' records. For example, the clinical data from first admission were screened for symptoms like hallucinations, delusions, ideas of reference or social withdrawal to examine if the described symptoms referred to the ICD-9 or ICD-10-criteria. Based on a consensus analysis, all subjects stayed in the study.

To track the patients we used phone numbers and addresses found in the patients' records of their first admission. Their homes were spread over a large area including Bavaria, northern Baden-Wuerttemberg, and southern parts of Hessen and Thuringia. Those patients whose addresses could not be verified after intensive search were dismissed from the study. 37 of the 86 subjects systematically targeted for enrolment could not be traced because they and their families moved to unknown domiciles. A review of death records revealed one case of death of unknown reason. Therefore, we tried to include 48 former patients to our study. 21 subjects (43.7%) refused to participate (8 women/13 men). This left 27 patients (56.3%) in the study (8 women/19 men).

Each participant or their legal representative signed a consent form after having the study explained to them.

### Variables and assessment instruments

To identify patients with the diagnosis of schizophrenia or schizoaffective disorder, two experienced clinicians reviewed the detailed hospital records from 1990 to 2000. For the assessment of characteristics of the first episode, ICD-9 and ICD-10 diagnoses for the first admission were taken from those records, as well as information about the anamnesis, symptomatology on admission, course of first inpatient treatment, family, and psychodiagnostic data (IQ and other test results).

After identifying the patients with schizophrenia or schizoaffective disorder (295, F20 or F25) diagnosis, the families or former patients were contacted in the next step by phone and were informed about the study. Then, we sent a cover letter, an information sheet and a questionnaire to the former patients (mail survey). The questionnaire consisted of several questions about psychopathological symptoms, further inpatient treatments, psychosocial functioning, and demographic characteristics like living situation, financial income, family situation, the state of education, and work. We also collected information about psychopathological and psychosocial outcome derived from semi-structured telephone interviews with patients and significant others before and after receiving the questionnaires. The semi-structured interview referred very closely to the items of the questionnaire.

For a standardized assessment of the schizophrenic symptoms and possible negative symptoms, two well established scales were included in the mail survey: the "Eppendorfer Schizophrenie-Inventar" (ESI) [21,22] and the "Allgemeine Depressions-Skala" (ADS) [23].

In our study, we applied the ESI total score and the ADS total score for statistical analysis. The ESI is a relatively new questionnaire for self-assessment of pre-psychotic and psychotic disturbances in several cognitive and perceptual areas. It was designed for diagnostic, therapeutic control and research purposes and is well validated. Compared to a well established instrument like the Frankfurt Complaint Questionnaire (FCQ), the ESI showed superior results regarding reliability and diagnostic validity. Several studies initiated to evaluate the ESI showed correlations to neuropsychological, psychopathological and anamnestic variables [22]. The total score ranges from 0 to 102. A cut-off value of 30 was established, as the authors found that only a small percentage (6.3%) of a non-schizophrenic sample had higher results. The ADS is a German scale for the assessment of depression, based on the "Center for Epidemiological Studies Depression Scale" (CES-D) [24]. In the case of the ADS, we used the short version, consisting of 15 items with a maximal total score of 45 and a cut-off value of 18.

In summary, we followed a multidimensional approach with standardized instruments to assess the patients' present state of outcome. All scales show satisfying reliability and validity.

### Statistical methods

First, we checked the variables regarding their distribution by using the Shapiro-Wilk-test and by evaluating the skewness and kurtosis. To compare dropouts with patients we followed up, we used chi-square tests for the categorical variable sex and Mann-Whitney-U-tests for continuous variables (age at first admission, duration of first inpatient treatment, follow-up-interval).

A comparison of the mean ESI- and ADS-scores between genders was drawn, using the Mann-Whitney-U-tests. To analyze a possible association between ESI- and ADS-scores, a Pearson correlation was performed.

For an exploratory analysis of associations with explanatory variables, the outcome variable for the degree of schizophrenic psychopathology (ESI-Score) was dichotomized via a median split. In order to investigate predictive factors, a logistic regression analysis was performed.

The significance level was fixed at α = 0.05. All statistical calculations were performed with SPSS 13.0.

## Results

### Follow-up sample characteristics

To date, questionnaire data of 27 former patients (8 women/19 men) could be collected. The mean time span between index hospitalization and follow-up was 13.4 years (SD 3.2 years). The mean age of participants at first admission was 15.5 years (SD 2.0 years) and 28.9 years (SD 4.0 years) at follow-up assessment. In four of the 27 cases (14.8%) the onset of schizophrenia was under the age of 14 years (VEOS). All patients with VEOS were males. Mean duration of first inpatient treatment was 81.9 days (SD 40.9 days) with a range of 35 – 206 days (Table 2).

**Table 2:** Characteristics of the follow-up sample

	**n**	**%**	**Mean**	**SD**
**Sex**				
female	8	29.6	-	-
male	19	70.4	-	-
**Age at first inpatient treatment**, years	-	-	15.4	2.03
**Age at follow-up**, years	-	-	28.85	3.96
**Length of follow-up interval**, years	-	-	13.37	3.17
**Duration of first inpatient treatment**, days	-	-	81.92	40.88
**Diagnoses**				
paranoid type	10	37	-	-
disorganized type	5	18.5		
catatonic type	9	33.3		
schizoaffective type	3	11.1		

The distribution of the diagnoses according to ICD-9- and ICD-10 criteria of the follow-up sample at first admission was as follows: 10 paranoid type (3 female/7 male), 5 disorganized type (2 female/3 male), 9 catatonic type (2 female/7 male), and 3 schizoaffective (1 female/2 male) (Table 2).

A dropout analyses conducted to compare the participants with the non-participants concerning gender difference, mean age at index hospitalization, duration of the first clinical admission due to schizophrenia, and length of follow-up-interval revealed no significant differences between the two groups (Table 3). Type of schizophrenia could not be investigated in the dropout analyses because of the small sample sizes.

**Table 3:** Dropout analyses: Comparison of participants and non-participants

	**Participants **(n = 27) Median (Range)	**Non-Participants **(n = 21) Median (Range)	**Significance**
**Age at first inpatient treatment**, years	16 (10 – 18)	16 (9 – 17)	n.s.
**Length of follow-up interval**, years	14 (7 – 19)	13 (7 – 18)	n.s.
**Duration of first inpatient treatment**, days	72.5 (35 – 206)	72 (5 – 164)	n.s.
**Sex ratio (female/male)**	8/19	8/13	n.s.

### Psychopathology

#### Course

At the follow-up assessment, 9 out of 27 cases (33.3%) were not re-hospitalized. 4 out of 27 participants (14.8%) were readmitted once after discharge. 14 former patients (51.8%) had at least two additional inpatient treatments after index hospitalization. Most of the former patients were still in outpatient treatment at follow-up assessment. 21 out of 27 (77.8%) had any form of an outpatient therapy: 18 were in contact with a psychiatrist, one visited a neurologist regularly, one a general practitioner and one person was looking for help through a homeopath. 17 patients received neuroleptic medication at follow-up. In 13 out of 17 cases the patients received more than one active substance.

#### Psychotic symptoms

The nature and severity of the symptoms at follow-up was assessed with ESI (schizophrenic symptoms) and ADS (symptoms of depression).

According to these self-rating scales, six patients (22.2%) had severe psychotic symptoms (ESI-score > 30). On average, it seemed that men showed more symptoms of schizophrenia at follow-up. But the difference between the ESI total score of men (median 16, range 1–50) and women (median 7.5, range 0 – 24) was not statistically significant (U = 50.5, N1 = 19, N2 = 8, p = 0.18, two-tailed).

#### Depressive symptoms

According to the ADS answers, one probands' questionnaire was not possible to evaluate and was therefore dismissed from data analysis. The data of the remaining 26 former patients showed that almost one third of them (8 out of 26 or 30.8%) revealed severe or moderate symptoms of depression. No differences between gender were found (U = 69.5, N1 = 18, N2 = 8, p = 0.89, two-tailed).

In addition to the standardized assessment of depressive symptoms, suicidal intentions were assessed. 10 out of 27 (37.0%) participants reported having tried to commit suicide or seriously thought about it.

To investigate if there is an association between the severity of psychotic (ESI total score) and depressive symptoms (ADS total score), a Pearson correlation was calculated but revealed no significant results (r = 0.30, N = 26, p = 0.13).

### Social outcome

#### Living situation

Concerning the living situation and independence of the former patients, the study revealed that almost half of participants were still living with their parents. Only a small part of the sample was living alone (14.8%) or together with their partner (3.7%). One third of the sample was not able to live independently and needed institutional support. No former patient was married (see Table 4 for detailed information).

**Table 4:** Living conditions, education and occupational situation 13.4 years after index hospitalization (n = 27; 8 women/19 men)

	**Sex**					
			
	Female		Male		**Total**	
	
	n	%	n	%	n	%
**Living situation at follow-up**						
Alone	2	25	2	10.5	4	14.8
With a partner	0	0	1	5.3	1	3.7
With parents	4	50	9	47.4	13	48.1
Sheltered or semi-sheltered living	2	25	7	36.8	9	33.3

**School education/graduation at follow-up**						
Special needs school	1	12.5	2	10.5	3	11.1
Grade 9 (Hauptschule)	4	50	8	42.1	12	44.4
Grade 10 (Realschule)	2	25	2	10.5	4	14.8
Grade 13 (Gymnasium)	0	0	3	15.8	3	11.1
Without qualification	1	12.5	4	21.1	5	18.5

**Occupational situation at follow-up**						
Unemployed	0	0	1	5.3	1	3.7
Unable to work	3	37.5	4	21.1	7	25.9
Occupied on a sheltered basis	4	50	9	47.4	13	48.1
Occupied on a non-sheltered basis	1	12.5	4	21.1	5	18.5
University study	0	0	1	5.3	1	3.7

#### Education

Before they were admitted to our clinic as schizophrenic, all patients attended school. For information, according to the German public school system, students attend one of three types of schools after completing four years of elementary school: a) Hauptschule that goes up to grade 9, b) Realschule, where students graduate after 10^th ^grade, and c) Gymnasium that continues up to grade 13 and prepares students for university. The majority of patients, twelve out of 27 (44.4%), graduated from the ninth grade at the "Hauptschule". Only three patients (11.1%) achieved the highest school graduation at the "Gymnasium" (see Table 4 for detailed results).

#### Occupation

An analysis of the occupational situation at the time of follow-up revealed that almost one half of the participants was employed in a sheltered setting. More than one fourth of the sample was unable to work at all. Only five of the former patients were occupied in the open market. One former patient was enrolled as a student at university (see Table 4 for detailed information).

### Risk factors

In order to analyse which of the variables present in the index episode were able to predict long-term outcome, a logistic regression analyses was performed. The dependent variable was the dichotomized ESI-Score. Subjects were divided into two groups: those with only a few schizophrenic symptoms (low ESI-score) and those with many schizophrenic symptoms (high ESI-score). Independent variables included in the model were: sex, age at first inpatient treatment because of schizophrenia, and duration of first admission. No significant associations were found, while a tendency towards a worse psychopathological outcome for male patients compared to female can be noted.

## Discussion

The preliminary data of our study describes course, psychopathological and psychosocial outcome of adolescent schizophrenia in 27 patients. Possible predictors of the life outcome were investigated.

### Strengths

As already mentioned the amount of long-term studies about VEOS and EOS is small compared to adult-onset schizophrenia, and most existing studies included a relatively limited number of patients. Only recent studies show more extensive sample sizes [10,16]. With a reassessment of 27 patients after a mean time span of 13 years, the investigation also has a considerable sample size and follow-up interval.

Advantageous of this study is the assessment of current schizophrenic symptoms in a mail survey by highly standardized instruments with satisfying reliability and validity. With the ESI we used an instrument that specifically measures psychotic symptoms. This allows us to estimate the psychopathological outcome even if the patients declined to participate in a planned face-to-face interview.

For the long-term evaluation of course and outcome of schizophrenia, Häfner and an der Heiden [19] demanded a multidimensional approach. In their opinion, it would be deficient to merely consider the global psychopathological state at follow-up. Therefore they distinguished between clinical, symptom related outcome and different aspects of the social outcome (e.g., family, living situation, education, and occupation). Thus we followed such a multidimensional approach of assessing the outcome of EOS.

### Limitations

Various methodological limitations are inherent to this study. As the study was designed as a retrospective follow-up of all schizophrenic patients admitted to the Department of Child and Adolescent Psychiatry in Wuerzburg between 1990 and 2000, our sample may only be representative for inpatient treatment. Compared to all patients, there might be a slight bias towards the range of more severe cases. On the other hand, it could also be possible that the group of patients willing to be reassessed might reveal less severity of symptoms compared to the group that was not agreeable to join the study.

Because of the retrospective assessment no reliable data concerning the treatment between first admission and follow-up could be collected.

Compared to other studies about the outcome of EOS, we had a relatively high dropout rate as many patients (44%) refused to participate.

Another limitation relates to the study design. In this study, only cross-sectional data were collected concerning the psychopathological and psychosocial outcome. Therefore, it is not possible to decide if a participant without severe psychopathological symptoms can be categorized as sane or remitted between two psychotic episodes. Another shortcoming deals with the mail survey as such. It is a fact that the self-report data of former schizophrenic patients about the psychopathological outcome are less reliable than rating scales done by experienced clinicians. We tried to minimize this limitation by collecting information about psychopathological and psychosocial outcome derived from semi-structured telephone interviews with patients and significant others before and after receiving the questionnaires.

Nevertheless, the main results of our study are in accordance to the results of other studies (see also Table 1). Overall, the psychopathological outcome of our study can be judged as poor. Every fifth patient displayed acute schizophrenic symptoms at follow-up and almost one third reported symptoms of depression. The unfortunate prognosis of EOS is emphasized by the high number of patients who are in outpatient treatment at follow-up. The high percentage of suicidality (37.0%) under this patient population also underlines the serious life impact of schizophrenia.

Evidence shows that former patients have severe problems in social life, implying that the early beginning schizophrenia impaired their psychosocial development. For example, almost half of the patients lived with their parents at follow-up. This number is twice as high as in the general population of young adults. For comparison, a general population survey [25] revealed that only one in four Germans between the ages of 18 and 30 lives with her/his parents. The current data correspond well with previous findings by Schmidt et al. [26]. In accordance to other studies [10], one third lived under assisted or semi-assisted living conditions. Only 5 out of 27 patients managed to live independently and autonomously.

Our study replicated the results by Fleischhaker and his colleagues [10] demonstrating that former schizophrenic patients achieve a lower educational degree on average and show higher school drop-out rates. For comparison, the general rate of achievement of different educational diplomas among Germans between the ages of 25 and 30 shows that only 3% had not graduated (18.5% of our sample) [27]. 69% graduate from grade 9 or 10 (59.2% of our sample) and 28% achieve the high-school equivalence "Gymnasium" (11.1% in our sample). As a consequence, educational problems led in many cases to occupational difficulties. Only a small percentage of former patients was able to find an occupation in the open market or study at university. Concerning the number of patients working on a sheltered basis or in a clinical setting (48.1%), this study compared well to prior findings by Fleischhaker et al [10].

As we also try to reassess former schizophrenic patients by face-to-face interviews (including PANSS, SPM, d2), a comparison of these data with the results of our mail survey is intended.

## Conclusion

Schizophrenia with early onset has an overall poor prognosis. Our study punctuates the findings of previous studies; the psychopathological and psychosocial outcome can generally be rated as poor for EOS. In contrast, adult long-term studies have often reported better outcomes concerning the schizophrenic symptoms and social adjustment. Our outcome supports Häfner's [6] opinion that an early onset leads to a dilapidated psychosocial outcome since many social developmental tasks are not yet completed by teenagers (e.g., the establishment of social roles, school graduation). Therefore, rehabilitation and social integration are imperative in the therapy of VEOS and EOS besides the necessary antipsychotic medication and psychotherapeutic support.

## Competing interests

The author(s) declare that they have no competing interests.

## Authors' contributions

All named authors have contributed substantially to the scientific process leading up to the writing of the paper and are entirely responsible for the scientific content of it: AR participated in the design, organization, and data collection of the study, performed the statistical analysis and drafted the manuscript. SK participated in the design, coordination and data collection of the study and helped to draft the manuscript. CMW participated in the design and organization of the study and helped to draft the manuscript. AW participated in the design and organization of the study and helped to draft the manuscript. All authors read and approved the final manuscript.
